# Long-Term Impact of Different Triple Combination Antihypertensive Medications on Blood Pressure Control, Metabolic Pattern and Incident Events: Data from the Brisighella Heart Study

**DOI:** 10.3390/jcm10245921

**Published:** 2021-12-17

**Authors:** Arrigo F. G. Cicero, Federica Fogacci, Elisabetta Rizzoli, Sergio D’Addato, Claudio Borghi

**Affiliations:** 1 Hypertension and Cardiovascular Risk Factors Research Center, Medical and Surgical Sciences Department, Sant’Orsola-Malpighi University Hospital, Via Albertoni 15, 40138 Bologna, Italy; federicafogacci@gmail.com (F.F.); elisabetta.rizzoli@unibo.it (E.R.); sergio.daddato@unibo.it (S.D.); claudio.borghi@unibo.it (C.B.); 2 IRCCS Policlinico S. Orsola-Malpighi di Bologna, 40138 Bologna, Italy

**Keywords:** Brisighella Heart Study, hypertension, triple combination antihypertensive medications, perindopril, amlodipine, indapamide

## Abstract

The aim of this study was to comparatively evaluate clinical, laboratory and hemodynamic effects on the long term of different triple combination antihypertensive medications in a well-characterized Italian cohort. We considered the data of a subset of Brisighella Heart Study (BHS) participants who were consecutively evaluated in three epidemiological surveys between 2012 and 2020. For the current analysis, we excluded normotensive subjects, patients treated with <3 or ≥3 antihypertensive drugs without taking angiotensin-converting enzyme (ACE) inhibitors, angiotensin II receptor blockers (ARBs), calcium-channel blockers (CCB) and/or thiazide/thiazide-like diuretics. The remaining participants were divided into three groups depending on whether they were treated with Perindopril/Amlodipine/Indapamide, ACE-inhibitors (other than perindopril)/CCBs/Thiazide or ARBs/CCBs/Thiazide, either with separate drugs or fixed pill combinations. A further group of age- and sex-matched volunteers was selected as control and included patients receiving other antihypertensive treatments. The long-term (8 years) effects of the different antihypertensive treatments were compared among the pre-defined groups. During the observation period, there was a trend towards increase in both systolic and diastolic blood pressure (BP) in all the investigated subgroups (*p* for trend <0.05), but in the subgroup of patients treated with Perindopril/Amlodipine/Indapamide, such increase was significantly lower than in the other groups (*p* < 0.05). The combination treatment with renin-angiotensin system (RAS) modulators, CCBs and thiazide/thiazide-like diuretics was associated with significantly lower diastolic BP (*p* < 0.05) and more strictly controlled lipid pattern than other triple combination of anti-hypertensive medications. Patients treated with Perindopril/Amlodipine/Indapamide did not experience any age-related increase in serum levels of total cholesterol. Moreover, during the follow up none of them developed type 2 diabetes, nor had a need for a greater number of antihypertensive drugs to improve BP control, mainly because of a more stable BP control. Based on our observations, combination treatment with RAS modulators, amlodipine and thiazides/thiazide-like diuretics is more effective than other triple antihypertensive medications for lowering the diastolic BP and has a better impact on serum lipids. Perindopril/Amlodipine/Indapamide is associated with more protective metabolic profile than any other considered combination antihypertensive medications.

## 1. Introduction

According to the results of a ground-breaking meta-analysis pooling data from 48 randomized clinical trials and 344,716 patients, a 5 mmHg reduction in systolic blood pressure (SBP) is able to decrease the risk for major cardiovascular (CV) events (MACE) by about 10%, irrespective of previous diagnosis of CV disease (CVD) and even in patients with normal to high-normal BP [1].

The higher BP pre-treatment levels, the more difficult is their reduction, so that patients with severe hypertension often require more than a drug to achieve an effective reduction in the risk of developing CVD [2]. Moreover, the number of drugs increases if optimal BP values are intended to be reached [3]. In this context, the latest international guidelines for the management of high BP recommend to start taking the polypill to improve low adherence to prescribed treatments, which is one of the major barriers to prevent CVD [4,5].

Findings from large-scale clinical trials are sound to support for the management of hypertension the use of combination therapy with renin-angiotensin system (RAS) inhibitors, calcium antagonists and a diuretic, when necessary. A meta-analysis of eight randomized clinical trials overall enrolling 20,451 hypertensive patients has recently highlighted the effectiveness of the association of RAS modulators and calcium antagonists to prevent MACE, which is greater than that expected for the same change in BP with alternative therapeutic approaches [6]. The most studied association of RAS modulators and calcium antagonists is the one combining perindopril and amlodipine, having been investigated in the Anglo-Scandinavian Cardiac Outcomes Trial-Blood Pressure Lowering Arm (ASCOT-BPLA) that enrolled 19,257 hypertensive patients and accumulated a total of 106,153 patient years of observation, before being prematurely stopped [7]. In this study, treatment with perindopril and amlodipine was associated with a reduced risk of fatal and non-fatal stroke [unadjusted hazard ratio (HR) = 0.77 95% Confidence Interval (CI) 0.66–0.89, *p* = 0.0003], total CV events and procedures (HR = 0.84 95%CI 0.78–0.90, *p* < 0.0001) and all-cause mortality (HR = 0.89 95%CI 0.81–0.99, *p* = 0.025) compared with atenolol and bendroflumethiazide [7]. These results were confirmed even more than 10 years after trial closure [8].

Even though the international guidelines recommend adding a third agent (usually a diuretic) to the RAS modulator and calcium antagonist to optimize BP control and CV risk [4,5], this recommendation is mostly supported by indirect evidence and small clinical trials [9,10]. For this reason, there is a need for further randomized clinical trials and real-world evidence comparing different triple combination of antihypertensive medications.

In this context, the aim of this analysis was to comparatively evaluate clinical, laboratory and hemodynamic effects on the long term of different triple combination antihypertensive medications in a well-characterized Italian cohort.

## 2. Materials and Methods

The Brisighella Heart Study (BHS) is a longitudinal epidemiological study which began in 1972. At the baseline, the BHS involved a randomized sample of 2939 Caucasian subjects (1491 men and 1448 women), aged 14–84 and free from CVD, that was representative of the entire population of Brisighella, a rural North Italian village. The study protocol was previously described in detail [11]. Briefly, participants were clinically evaluated at baseline and every 4 years thereafter, by collecting demographic and clinical data and laboratory samples. Mortality and morbidity data, as well as the incidence of the main CV risk factors, were recorded throughout the entire study [12].

The study protocol was approved by the Institutional Ethical Board of the University Hospital of Bologna (AVEC; Code: BrixFollow-up_1972-2024), and it was performed in accordance with the ethical standards laid down in the 1964 Declaration of Helsinki and its later amendments. All involved subjects signed an informed consent form prior to their inclusion in the study.

Every subject was evaluated with a detailed assessment of personal and family history (with particular attention to lifestyle and dietary habits, smoking status and pharmacological treatments), a physical examination (including anthropometric data), resting BP and heart rate. Fasting blood samples and 12-lead electrocardiograms (Minnesota-coded) were also collected [13]. Waist circumference (WC) was measured as the narrowest body diameter between arcus costarum and crista iliaca. Height was evaluated with the person standing erect, bare feet together and eyes directed straight ahead. Weight was measured twice, and the average measure was used. Body mass index (BMI) was calculated as weight in kilograms divided by height in meters squared (kg/m^2^). Systolic (SBP) and diastolic (DBP) BP were measured three times at 1 min interval with a standard sphygmomanometer, while the patient was seated and after 5 min of quiet rest. Three BP measurements were averaged and used as study variable [14]. Standard 12-lead resting ECG recordings were performed using a Marquette MAC 12 electrocardiograph (ECG) (Marquette Medical Systems, Inc., Milwaukee, WI, USA) with signals sampled at 250 Hz per channel. A representative P-QRS-T cycle was then derived by selective averaging using the Dalhousie ECG Analysis Program [15]. The automated diagnosis of the ECG software was blindly confirmed by a trained cardiologist. Left ventricular hypertrophy (LVH) was calculated according to the Sokolow–Lyon formula, as recommended by current guidelines [5], and the diagnosis was further confirmed by the R-wave voltage in lead aVL, that seems to present a greater diagnostic ability in detecting LVH at a population level [16].

Laboratory analyses were carried out on venous blood from the basilic vein. Subjects were fasted for at least 12 h at the time of sampling. The following laboratory parameters were sampled with standardized methods by trained personnel [17]: fasting plasma glucose (FPG), total cholesterol (TC), triglycerides (TG), high-density lipoprotein cholesterol (HDL-C), LDL-C, aspartate aminotransferase (ALT), alanine aminotransferase (AST), gamma-glutamyl-transferase (GGT), creatinine, serum uric acid (SUA) and creatinine phosphokinase (CPK). Glomerular filtration rate (eGFR) was estimated by the Chronic Kidney Disease Epidemiology Collaboration (CKD-EPI) formula [18].

### 2.1. Subject Selection

**Figure 1:**
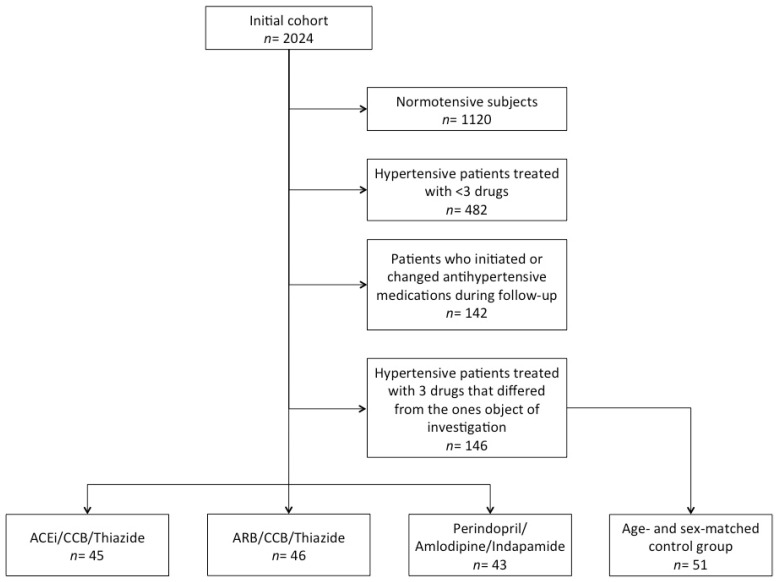
Flow-chart resuming the selection criteria applied to the full BHS cohort to select the sub-cohort.

For the purpose of this analysis, we considered data of a subset of Brisighella Heart Study (BHS) participants who were consecutively evaluated in three epidemiological surveys between 2012 and 2020. We excluded normotensive subjects, patients treated with <3 or ≥3 high BP medications without taking angiotensin-converting enzyme (ACE) inhibitors, angiotensin II receptor blockers (ARBs), calcium-channel blockers (CCB) and/or thiazide/thiazide-like diuretics. The remaining participants were divided into three groups depending on whether they were treated with Perindopril/Amlodipine/Indapamide, ACE-inhibitors (other than perindopril)/CCBs/Thiazide or ARBs/CCBs/Thiazide, either with separate drugs or fixed pill combinations (Figure 1). A further group of age- and sex-matched volunteers was selected as control and included patients receiving other triple antihypertensive medications (Figure 1).

Incident diabetes was defined as two successive diagnostic FPG levels >125 mg/dL and/or initiation of oral antidiabetic drugs or insulin during follow-up. MACEs included incident heart failure, atrial fibrillation, acute coronary syndrome, coronary revascularization, stroke, incident claudicatio or acute limb ischemia, or its recidivisms, or sudden death.

### 2.2. Statistical Analysis

Considering that the group treated with Perindopril/Amlodipine/Indapamide association was relatively less represented among the considered ones, we carried out a post hoc power analysis comparing the rate of BP control of this subgroup with the other ones.

A full descriptive analysis was performed according to background antihypertensive treatment for all the parameters collected during the 2012, 2016 and 2020 population surveys. Continuous variables were reported as mean ± standard deviation. Categorical data were expressed as number (percentage). The Kolmogorov–Smirnov test was performed to assess normality for continuous variables. A *p* for trend was calculated by the analysis of variance (ANOVA) for repeated measures or Friedman test. Generalized estimating equation (GEE) models were applied to examine the associations between type of antihypertensive treatment and changes in the collected parameters. A chi-squared test was carried out to compare event incidences. A two-tailed *p* level less than 0.05 was considered to be statistically significant for all tests. Statistical analysis was performed with Statistical Package for Social Sciences (SPSS) version 25.0 for Windows (IBM Inc., Chicago, IL, USA).

The study report is compliant to the STROBE (Strengthening the Reporting of Observational Studies in Epidemiology) guidelines, and to the broader EQUATOR (Enhancing the QUAlity and Transparency Of health Research) guidelines [19].

## 3. Results

The main characteristics of the patients included in the analysis are listed in Table 1.

At the baseline, the percentage of people reaching a BP < 140/90 mmHg was 68% among ACE-inhibitors/CCBs/Thiazide-treated subjects, compared with 61% among subjects treated with ARBs/CCBs/Thiazide, compared with 71% among subjects in treatment with Perindopril/Amlodipine/Indapamide and compared with 59% among subjects treated with any other considered combination antihypertensive medications (*p* < 0.05).

At the end of the observation, the percentage of people reaching a BP < 140/90 mmHg was 65% among ACE-inhibitors/CCBs/Thiazide-treated subjects, compared with 59% among subjects treated with ARBs/CCBs/Thiazide and compared with 69% among subjects in treatment with Perindopril/Amlodipine/Indapamide, compared with 58% among subjects treated with any other considered combination antihypertensive medications (*p* < 0.05).

During the observation period, despite the treatment, there was a trend towards increase in both systolic and diastolic BP in all the investigated subgroups (*p* for trend 2012–2016–2020 <0.05), but in the subgroup of patients treated with Perindopril/Amlodipine/Indapamide, such an increase was significantly lower than in the other groups (*p* < 0.05).

The laboratory analyses demonstrate that from 2012 to 2020, TC increased in all subgroups except in the subgroup of patients treated with Perindopril/Amlodipine/Indapamide. LDL-C and TG significantly increased in all subgroups (*p* for trend 2012–2016–2020 <0.05). However, subjects with ACE-inhibitor (Perindopril included)-based treatment had levels of LDL-C and TG significantly lower than subjects included in the control group (*p* < 0.05).

SUA increased only in the ACE-inhibitors/CCBs/Thiazide group (*p* for trend 2012–2016–2020 <0.05), whereas eGFR decreased in all subgroups (*p* for trend 2012–2016–2020 <0.05).

In patients without electrocardiographic-LVH (ECG-LVH) at baseline, incident ECG-LVH was 8.4% among ACE-inhibitors/CCBs/Thiazide-treated subjects, compared with 6.9% among the ARBs/CCBs/Thiazide-treated subjects, compared with 4.2% among the Perindopril/Amlodipine/Indapamide-treated subjects and compared with 9.8% among the control group (*p* for the trend 2012–2016–2020 <0.05).

Over a follow-up period of 8 years, the proportion of subjects who developed type 2 diabetes were 2 (4.4%), 2 (4.3%) and 0 (0%) among ACE-inhibitors/CCBs/Thiazide-, ARBs/CCBs/Thiazide- and Perindopril/Amlodipine/Indapamide-treated patients, respectively. The proportion of subjects who had a need for a greater number of antihypertensive drugs to improve BP control were 2 (4.4%), 3 (6.5%) and 0 (0%) among ACE-inhibitors/CCBs/Thiazide-, ARBs/CCBs/Thiazide- and Perindopril/Amlodipine/Indapamide-treated patients, respectively. The proportion of subjects who experienced MACEs among ACE-inhibitors/CCBs/Thiazide-, ARBs/CCBs/Thiazide- and Perindopril/Amlodipine/Indapamide-treated patients were 4 (8.8%), 4 (8.6%) and 2 (4.6%) patients, respectively. In the control group, we recorded 3 (5.9%) cases of onset diabetes and 8 MACEs (15.7%); moreover, 5 subjects (9.8%) had a need for a greater number of antihypertensive drugs to improve BP control.

Finally, the proportion of subjects who died because of non-CV reasons were 3 (6.6%), 2 (4.3%) and 2 (4.6%) patients in the ACE-inhibitors/CCBs/Thiazide, ARBs/CCBs/Thiazide, Perindopril/Amlodipine/Indapamide groups and 3 (5.9%) in the control group (*p* > 0.05), respectively.

## 4. Discussion

In this study, on the long-term, combination treatment with RAS modulators, calcium antagonists and thiazide/thiazide-like diuretics was associated with better control of DBP and of lipid pattern than other triple combination antihypertensive medications. Patients treated with Perindopril/Amlodipine/Indapamide did not experience any age-related increase in serum levels of TC. Moreover, during the follow-up they neither developed type 2 diabetes nor had a need for a greater number of antihypertensive drugs to improve BP control. These results are in line with previous findings from large clinical trials, showing no negative metabolic effects of the triple-drug therapy Perindopril/Amlodipine/Indapamide [20,21].

During follow-up, the group treated with Perindopril/Amlodipine/Indapamide did not show an increase in SUA, contrarily to what observed in the ACE-inhibitors/CCBs/Thiazide-treated group. This was expected, because of the indapamide neutral effect on urate excretion, in contrast with the SUA increasing effect of thiazide diuretics [22]. SUA also did not increase in the ARBs/CCBs/Thiazides-treated group, probably because of the known positive effect of some ABRs on SUA levels [23].

Moreover, the known positive long-term impact of Perindopril/Amlodipine/Indapamide on ECG-LVH [24] was confirmed in our cohort, we observed a significantly lower incidence of LFH in Perindopril/Amlodipine/Indapamide-treated subjects in comparison with the other subgroups.

The present analysis has some limitations that have to be noted. First, application of strict inclusion criteria significantly reduced the sample size, which, however, reflects the real-world use of the investigated combination antihypertensive drugs. Moreover, the age-dependent slight rise in BP is likely to have been partially affected by antihypertensive drug dose increases over the years. On the other side, in the current analysis, we evaluated the effect of triple-combination antihypertensive drugs usually assumed as polypill containing two medications and a free drug. However, the use of a single fixed pill combination would have increased the effectiveness of the treatments by improving patients’ compliance [25,26]. Another limitation is the lack of information on electrolytes—including potassium—whose serum levels are often affected by diuretics. Furthermore, echocardiography was not included in the BHS protocol. For this reason, LVH was defined by ECG criteria, with a relatively low sensitivity in the diagnosis [27].

It should also be also taken into account that the BHS included an educational intervention aimed at improving the dietary habits and behaviors of all participants [28]. This has certainly reduced the incidence of MACE—that was in general low—, and also the probability of observing a significant difference among the considered population groups. Finally, the number of incident events was not enough to estimate the survival function and draw a Kaplan–Meier plot.

Despite these limitations, the present study describes the long-term health outcomes of different triple combination antihypertensive drugs in a well-characterized population cohort. The data on hypertension control rate are in line with the Italian and European ones [29]. Our study further supports the indication of the most recent guidelines for cardiovascular prevention in clinical practice about the use of multiple antihypertensive drugs to achieve an effective cardiovascular disease risk reduction. In particular, they confirm the efficacy and safety of ACE-inhibitors and calcium antagonists [4,5,30].

In conclusion, based on our observations, combination treatment with RAS modulators, amlodipine and thiazides/thiazide-like diuretics is mildly but significantly more effective than other combination of antihypertensive drugs for lowering BP, and has a better impact on serum lipids. Perindopril/amlodipine/indapamide is associated with a better metabolic profile than any other considered combination of antihypertensive medications.

## Figures and Tables

**Table 1:** Clinical and laboratory characteristics of the hypertensive patients included in the Brisighella Heart Study sub-analysis.

Parameters	ACE-Inhibitors/CCBs/Thiazide (*n* = 45; M = 23, W = 22)			ARBs/CCBs/Thiazide (*n* = 46; M = 20, W = 23)			Perindopril/Amlodipine/Indapamide (*n* = 43; M = 21, W = 24)			Other Treatments (*n* = 51; M = 28, W = 31)		
	2012	2016	2020	2012	2016	2020	2012	2016	2020	2012	2016	2020
Age (years)	56 ± 3	60 ± 4	64 ± 4 *	57 ± 4	61 ± 4	65 ± 4 *	56 ± 4	60 ± 3	64 ± 4 *	56 ± 3	60 ± 3	64 ± 4 *
WC (cm)	96 ± 6	96 ± 7	97 ± 7	95 ± 7	95 ± 7	97 ± 7	94 ± 7	95 ± 6	97 ± 6	94 ± 4	96 ± 6	98 ± 6
BMI (kg/m^2^)	27.8 ± 2.1	27.8 ± 2.2	27.9 ± 2.2	27.6 ± 2.2	27.6 ± 2.1	27.9 ± 2.2	27.4 ± 2.3	27.6 ± 2.0	27.8 ± 2.3	27.4 ± 2.2	27.8 ± 2.1	27.9 ± 2.4
SBP (mmHg)	145 ± 6	146 ± 5	147 ± 5 *	145 ± 5	147 ± 6	148 ± 7 *	142 ± 4	143 ± 5	144 ± 6 *	147 ± 6	149 ± 6	149 ± 10 *
DBP (mmHg)	73 ± 3	75 ± 2	76 ± 3 *^	74 ± 3	75 ± 3	77 ± 4 *^	73 ± 2	73 ± 3	75 ± 3 *^	75 ± 4	77 ± 5	80 ± 4 *
PP (mmHg)	72 ± 3	71 ± 3	71 ± 3	71 ± 3	72 ± 3	71 ± 4	69 ± 2	70 ± 3	69 ± 3	72 ± 3	72 ± 4	69 ± 4
MAP (mmHg)	97 ± 4	98 ± 4	98 ± 4	97 ± 3	98 ± 4	98 ± 5	96 ± 2	96 ± 3	97 ± 3	98 ± 4	99 ± 4	99 ± 4
HR (bpm)	65 ± 6	64 ± 6	67 ± 7	64 ± 6	65 ± 7	65 ± 7	65 ± 8	66 ± 8	66 ± 7	66 ± 5	66 ± 7	68 ± 7
TC (mg/dL)	201 ± 14	205 ± 12	211 ± 15 *^	199 ± 13	204 ± 15	212 ± 12 *^	196 ± 11	199 ± 12	202 ± 12 ^°	212 ± 13	216 ± 13	229 ± 15 *
TG (mg/dL)	119 ± 24	124 ± 22	129 ± 23 *^	122 ± 16	127 ± 21	132 ± 19 *^	115 ± 16	119 ± 17	120 ± 19 *°^	125 ± 21	131 ± 25	149 ± 24 *
HDL-C (mg/dL)	42 ± 3	42 ± 3	41 ± 3	43 ± 3	42 ± 3	41 ± 3	44 ± 2	44 ± 2	43 ± 3	41 ± 2	42 ± 2	40 ± 3
LDL-C (mg/dL)	135 ± 11	138 ± 11	144 ± 13 *^	132 ± 10	137 ± 11	145 ± 10 *	129 ± 9	131 ± 10	135 ± 10 *°^	146 ± 11	148 ± 12	159 ± 13 *
FPG (mg/dL)	97 ± 7	99 ± 8	100 ± 9	95 ± 7	95 ± 9	98 ± 9 *	94 ± 5	94 ± 7	96 ± 8 *^	98 ± 7	100 ± 6	102 ± 8 *
SUA (mg/dL)	5.5 ± 0.6	5.7 ± 0.8	6.0 ± 0.6 *	5.4 ± 0.4	5.6 ± 0.5	5.6 ± 0.7	5.3 ± 0.3	5.5 ± 0.3	5.6 ± 0.4	5.8 ± 0.4	5.8 ± 0.7	5.9 ± 0.9
eGFR (mL/min)	75.5 ± 5.4	72.1 ± 4.9	69.3 ± 4.2 *	76.2 ± 5.3	72.7 ± 4.8	70.2 ± 4.4 *	77.8 ± 5.1	75.7 ± 4.8	72.3 ± 4.9 *	74.2 ± 5.1	70.4 ± 5.2	64.9 ± 4.9 *

* *p* for trend 2012–2016–2020 <0.05; ° *p* < 0.05 versus all other subgroups, ^ *p* < 0.05 versus other treatment. BMI = Body mass index; DBP = Diastolic blood pressure; eGFR = Estimated glomerular filtration rate; FPG = Fasting plasma glucose; HDL-C = High-density lipoprotein cholesterol; HR = Heart rate; LDL-C = Low-density lipoprotein cholesterol; M = Men; MAP = Mean arterial pressure; *n* = Number of subjects; PP = Pulse pressure; SBP = Systolic blood pressure; SUA = Serum uric acid; TC = Total cholesterol; TG = Triglycerides; W = Woman; WC = Waist circumference.

## Data Availability

Data supporting findings of this analysis are available from the University of Bologna. Data are available from the authors with the permission of the University of Bologna.
