# Correction: Cicero et al. Long-Term Impact of Different Triple Combination Antihypertensive Medications on Blood Pressure Control, Metabolic Pattern and Incident Events: Data from the Brisighella Heart Study. *J. Clin. Med.* 2021, *10*, 5921

**DOI:** 10.3390/jcm11237109

**Published:** 2022-11-30

**Authors:** Arrigo F. G. Cicero, Federica Fogacci, Elisabetta Rizzoli, Sergio D’Addato, Claudio Borghi

**Affiliations:** 1 Hypertension and Cardiovascular Risk Factors Research Center, Medical and Surgical Sciences Department, Sant’Orsola-Malpighi University Hospital, Via Albertoni 15, 40138 Bologna, Italy; 2 IRCCS Policlinico S. Orsola-Malpighi di Bologna, 40138 Bologna, Italy

## Text Correction

There was an error in the original publication [1]. In the abstract and results sections, the follow-up was defined as 12-years instead of 8-years.

A correction has been made in the abstract and in the result section (eighth paragraph): the number “12” was changed with “8”. 

The authors apologize for any inconvenience caused and state that the scientific conclusions are unaffected. This correction was approved by the Academic Editor. The original publication has also been updated.
